# Culture’s building blocks: investigating cultural evolution in a LEGO construction task

**DOI:** 10.3389/fpsyg.2014.01017

**Published:** 2014-09-12

**Authors:** John J. McGraw, Sebastian Wallot, Panagiotis Mitkidis, Andreas Roepstorff

**Affiliations:** ^1^Interacting Minds Centre, Department of Culture and Society, Aarhus UniversityAarhus, Denmark; ^2^Center for Advanced Hindsight, Social Science Research Institute, Duke UniversityDurham, NC, USA; ^3^Interdisciplinary Centre for Organizational Architecture, School of Business and Social Science, Aarhus UniversityAarhus, Denmark

**Keywords:** cultural evolution, cultural transmission, joint action, joint attention, shared intentionality, materiality, path dependence, schema theory

## Abstract

One of the most essential but theoretically vexing issues regarding the notion of culture is that of cultural evolution and transmission: how a group’s accumulated solutions to invariant challenges develop and persevere over time. But at the moment, the notion of applying evolutionary theory to culture remains little more than a suggestive trope. Whereas the modern synthesis of evolutionary theory has provided an encompassing scientific framework for the selection and transmission of biological adaptations, a convincing theory of cultural evolution has yet to emerge. One of the greatest challenges for theorists is identifying the appropriate time scales and units of analysis in order to reduce the intractably large and complex phenomenon of “culture” into its component “building blocks.” In this paper, we present a model for scientifically investigating cultural processes by analyzing the ways people develop conventions in a series of LEGO construction tasks. The data revealed a surprising pattern in the selection of building bricks as well as features of car design across consecutive building sessions. Our findings support a novel methodology for studying the development and transmission of culture through the microcosm of interactive LEGO design and assembly.

## INTRODUCTION

Natural selection has proven to be a uniquely successful scientific paradigm. By identifying the basic processes through which organisms change, Darwin (1859) established a research program that has not only revolutionized the study of life, but has provided a template for what a comprehensive model of transformation over time ought to look like. And with the additional refinements and achievements of the modern evolutionary synthesis, many of the subtler mechanisms, including the way that biological information is genetically transmitted, have yielded to scientific inquiry and experimentation (Dobzhansky, 1937; Huxley, 1942; Mayr, 1942). Moreover, because evolution—for the most part—subsumes all aspects of biological life, it has been used not only as an explanation of changes in biological *form*, but of the *behavior* of organisms (Darwin, 1890; Lorenz, 1937; Tinbergen, 1951). Indeed, some theorists believe that the mysteries of human behavior, and the achievements of human societies, may ultimately find their explanations in rigorous applications of evolutionary theory to patterns of human interaction, potentially explaining culture itself (Wilson, 1975; Dawkins, 1976; Sperber, 1996). But in spite of many attempts to adapt ideas from biological evolution to the study of culture, beginning soon after the publication of Darwin’s magnum opus (Spencer, 1864; Galton, 1869; Haeckel, 1900), the preliminary approaches have, as of yet, failed. This includes even the impressively nuanced models of such 20th century scholars as Steward (1955) and Parsons (1966). But if theorists of society have had the archetype of biological evolution to inspire them for so long, why have they come up short in their attempts to achieve something similar for culture? Is culture qualitatively different than biology, so that attempting to create an “evolutionary theory” of culture is non-sensical, or a mere metaphor? In agreement with a growing number of scholars (Boyd and Richerson, 2005; Mesoudi, 2011; Sterelny, 2012), we hold that many of the basic processes which undergird the evolutionary theory of life apply to culture as well. Inspired by Darwin’s meticulous study of details, we hypothesize that an evolutionary theory of culture will develop through careful observations of the smallest phenomena that can still be called “cultural.” And just as Darwin gradually came to an understanding of natural selection by noting tiny differences among barnacles, finches, and other creatures, an understanding of the evolutionary processes of culture will likely derive from particularistic studies of culture’s “building blocks.”

We identify these building blocks as skills that human beings are uniquely predisposed to develop during infancy and childhood, but *only* through engaging others in richly scaffolded^1^ cultural contexts (Luria, 1976; Vygotsky, 1978; Hobson, 2002; Rogoff, 2003; Reddy, 2008). We use the term “skill” to indicate a theoretical framework of human behavior as constituted by capacities of relationality to people and things in pre-existing, culturally engineered environments. Additionally, the term skill suggests a capacity that: (1) is developed; (2) never achieves a final state of enskilment, but is essentially determined by the continued exercise of the skill; and (3) depends upon all earlier uses of the skill, that is say, a skill always has a “history.” It is upon the foundation of our “skills for intersubjectivity” and our “skills for interobjectivity” that culture is built. And to crib from Darwin (1859, 490): “…from so simple a beginning endless forms most beautiful and most wonderful have been, and are being, evolved.” But just as natural selection was not apparent before Darwin’s studies, so cultural evolution remains little more than a tantalizing mirage until its processes are rendered visible. The theory of natural selection was developed, and continues to be refined, by studying physical organisms as well as their ancestors’ fossilized remains. In order to make culture visible, its *processes* must be operationalized in particular forms of interaction and materialized in *products* of those interactions; the object of study must first be an “object” before an empirical science can truly begin. We attempted to accomplish this very thing in our quasi-naturalistic joint action experiment.

**FIGURE 1:**
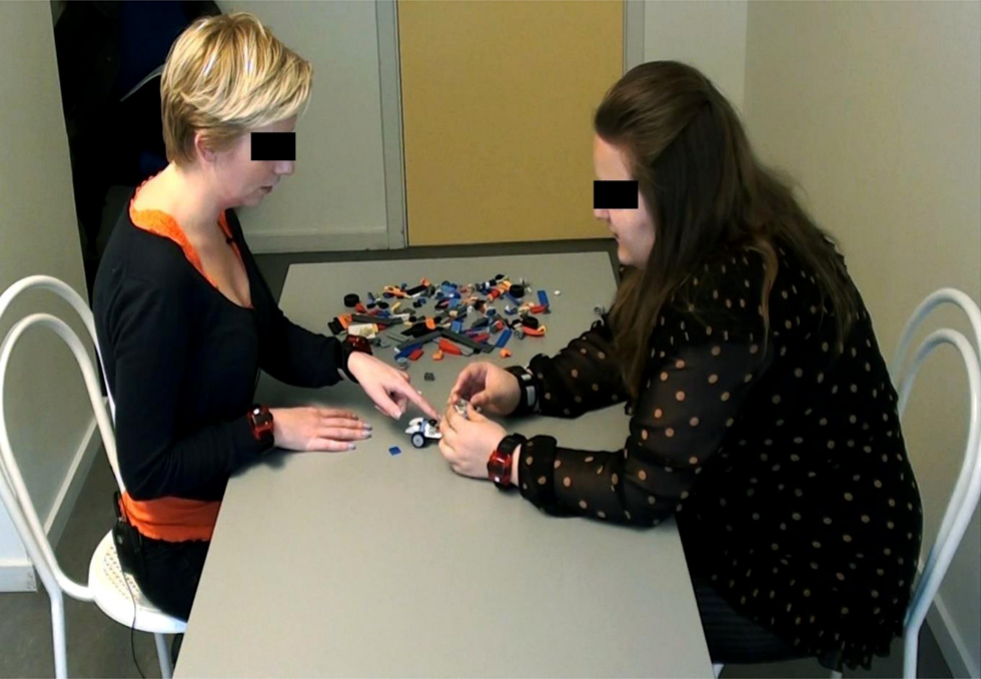
**Photo of participants building a car model**.

In the study, pairs of participants were required to construct four model cars using LEGO®; building bricks (see **Figure 1**). The pairs constructed their models in consecutive 10 min building sessions and employed distinctive “modes of interaction” during each of these sessions: egalitarian cooperation (EC), turn-taking (TT), and hierarchical cooperation (HC). At the beginning of each of the four sessions, participants were given written instructions for one of these modes of interaction. For EC, participants were directed to go about building their car however they saw fit. For TT, participants took turns in designing the car: one person offered a design suggestion while the other aided in constructing that feature and then they would reverse roles. For HC, one participant served as the “director” in charge of design decisions throughout the entire session. Upon ending the first HC session, participants reversed roles in the very next session. After each 10 min building session, we collected the car and remaining LEGO bricks and supplied the pairs with an identical set of building bricks at the beginning of the following session. The cars themselves served as our primary source of data, as described in more detail below.

### THEORETICAL FRAMEWORK

Dynamics representative of larger cultural processes are at work in this experimental task. The design and assembly of a model car is a type of joint action, in which “two or more individuals coordinate their actions in space and time to bring about a change in the environment” (Knoblich et al., 2011). Much of what goes by the name “culture” could be defined in the same way (see Risjord, 2012). Additionally, these actions are “embodied practices of mind” (Gallagher, 2005, 206–236) whose proper unit of analysis is the coordinated dyad engaged in “participatory sense-making” (De Jaegher and Di Paolo, 2007). As Risjord (2007, 414) observes: “Culture is neither a psychological phenomenon nor some kind of abstraction from individuals. It is the social interactions themselves, perfectly public and observable, yet distinct from any individual participant.” Culture may be thought of as some arrangement of interlocking joint actions that build up from two people to larger and larger groups. Joint action relies on joint attention, the mutual attendance to an object indexed by such things as gaze following (Tomasello, 2008), and shared intentionality, the capacity to develop shared goals and coordinate actions toward the achievement of those goals (Tomasello and Carpenter, 2007; Gallotti and Frith, 2013). Additionally, orchestrating these skills in model car construction is an example of collaborative engagement in which “agents share goals and action plans manifested in a joint intention” (Tomasello et al., 2005). Numerous scholars have suggested this ability for shared intentionality and cooperation to be essential for culture (Tomasello, 1999b; Schönpflug, 2008; Boyd and Richerson, 2009).

How could simple tasks performed in such short time scales reveal anything substantial about cultural evolution? Joint actions are forms of “microgenesis” (Werner, 1957; Rosenthal, 2004; Sinha, 2005, 1553), that is, developmental processes unfolding in “real time.” And whether demonstrated through “triadic interactions” between infants and caregivers (Trevarthen and Hubley, 1978), the assembly of LEGO models by adults (Clark and Krych, 2004; Bjørndahl et al., 2014), or troops coordinating their movements on a battlefield—all exemplify microgenesis. Longer term processes of cultural evolution, although undoubtedly more complex, are built from microgenetic actions. Just as a biologist might focus on macroevolution or microevolution (Filipchenko, 1927; Dobzhansky, 1937), both exhibit the identical processes of natural selection; the model of selection behind the rise of the dinosaurs and the model of selection behind the antibiotic resistance of a given species of Staphylococcus are one and the same. So while studying long term changes of social organization is a viable means to investigate the topic, we must turn to real time interactions if we are to build an experimental science of cultural evolution.

#### Skills for intersubjectivity

Intersubjectivity has been defined as “the sharing of experiential content (e.g., feelings, perceptions, thoughts, and linguistic meanings) among a plurality of subjects” (Zlatev et al., 2008). It is marked by such things as shared emotions (Michael, 2011), empathy (Scheler, 1954; Zahavi, 2008), and “resonance systems” which lead us to experience, in some partial way, the “feeling” of an action when watching someone else perform the action (Gallese, 2001; Gallagher, 2008). Many of the processes that get bundled together in the term “intersubjectivity” begin to develop during infancy as a set of skills: “…capabilities of action and perception of the whole organic being (indissolubly mind and body) situated in a richly structured environment. As properties of human organisms, skills are thus as much biological as cultural” (Ingold, 2000, 5). The development of these skills emerges from interacting with older, more competent humans engaged in *task-oriented patterns of practice*, even if these practices are “mere play” (Hobson, 2002, 42; Di Paolo et al., 2010, 72–78).

Trevarthen and Hubley (1978) discussed the easy imitation of smiles, cooing, and interpersonal gaze by young infants as “primary intersubjectivity” which begins to give way, around 9 months of age, to “secondary intersubjectivity.” Discussing this more recursive form of intersubjectivity, Hobson (2002, 61) notes: “Clearly, personal relations are not just about exchanging smiles and coos and other endearing or not-so-endearing gestures with someone else. They are also about sharing experiences of things. Personal relations are about connecting with someone else and making reciprocal emotional contact, but also about exchanging points of view, or agreeing and disagreeing about this or that, or sharing jokes. If we can clarify how infants engage with someone else so that communication is *about* a third object or outside event, then we may draw closer to seeing how they come to think about things.” Infants begin to demonstrate secondary intersubjectivity by participating in “triadic interactions” which involve “a referential triangle of child, adult, and the object or event to which they share attention” (Tomasello, 1999a, 62). A classic task of this kind is the rolling of a ball back-and-forth between child and caregiver. At this young age, then, humans begin to fluidly engage in practices that introduce them to the conventionalized uses and meanings of objects. Enculturation, the long term process by which a person acquires the requisite languages, skills, and sensibilities of the groups to which she belongs (Wexler, 2006; Kiverstein and Farina, 2011; Lende and Downey, 2012), depends on forms of social cognition that develop from engaging in playful activities of this sort. It may be significant to note that human infants *enjoy* the processes and practices of becoming a cultural being. Creating productive research programs to investigate cultural evolution ought to look for these sorts of enjoyable activities as indices of being on the right path.

But the attraction to games and then stories that so marks early stages of development are also forms of “serious play.” From these activities norms, rules, and values are introduced to the child who quickly begins to embody his particular culture’s mores (Sinha, 2009, 174–176). With these normative engagements with others and with objects, the child also begins to intelligently observe and act on regularities in the environment, a process called *schematization* (see Piaget, 1952). Schemas are memories that an individual develops for recurrent features of the world which are sufficiently open and flexible to apply to “sets” or “categories” rather than to idiosyncratic items (Bartlett, 1932; Rumelhart, 1980; McGraw, 2007). For an English-speaking person in the contemporary globalized world, schemas would be typical for such things as “trees,” “buildings,” and “flags,” but probably not for “cyclotrons,” “halberds,” or “transepts.” A schema for a car, for instance, would include basic characteristics like “four-wheeled vehicle,” “possesses an enclosed space for driver and passengers,” and a host of typical components (e.g., steering wheel, windshield, headlights). The fact that people would use a modifier before the term to identify items uncommon for the set (e.g., three-wheeled car, flying car, solar car) suggests the importance of common features in the development and consolidation of schemas.

While schemas are routinely demonstrated in our daily interactions, trying to find them in language presents many challenges that researchers have been wrestling with for decades (D’Andrade, 1995; Shore, 1996; Quinn, 2005). Sinha (2005, 1538) suggests an alternative approach very much in line with our study: “…cognitive and cultural schemas find material realization—are embodied—in the artifacts of material culture; and the way in which such artifacts are themselves embedded in culturally appropriate, normative structures of action and interaction. In this perspective, mind is socially distributed between people, and mental processes are supported by objects which embody and represent them. Cognition extends beyond the individual; embodiment goes beyond the skin.” Searching for schemas in the physical world seems eminently preferable to inferring them from language since investigating the materialization of schemas affords a more quantitative and empirical approach, as we demonstrate in the analysis below.

#### Skills for interobjectivity

Unfortunately, the shadow of Descartes still looms large; just as understanding mind apart from body is now perceived to be a philosophical blunder, so trying to understand the social and the cultural without considering its material basis revisits a distressingly common “category mistake” (Ryle, 1949). Though culture is made up of bodies, places, and things, many discussions about culture would lead one to think it was composed of abstract forces alone (see Latour, 2005). However, reflecting on people and social forces without consideration of their material aspects and accompaniments reveals itself to be an impoverished substitute: try to imagine Roman Catholicism without Bibles, churches, communion wafers, monasteries, crucifixes, tombs, or Rome. Culture is a particular coordination among “things” in the world, including but not limited to bodies, places, structures, and technologies. Coordination among these various things, through languages, customs, and rituals, does not exist apart from them. Even the notion of a culture apart from the things in the world that make it up turns out to be empty of content. One of the goals of this article is to highlight the ineliminable materiality that goes along with culture and, consequently, with cultural transmission (Sinha and Rodríguez, 2008; Sinha, 2009). Discussions of cultural transmission must take into account the fact that the social and the material are necessarily linked, even if many scholars have seen the latter as mere effect or consequence of the former. In fact, the social and the material co-constitute one another, so that one cannot reasonably discuss one without the other (Latour, 1996; Miller, 1998, 2005; Malafouris, 2013). Additionally, because of the differences, particularly in time scale, between behaviors, bodies, and artifacts, each of these employs distinctive processes of cultural transmission. Nevertheless, robust forms of cultural transmission are demonstrable in each of these activities and structures, and across their varying time scales, from the immediate effects of imitative learning between children and caregivers to the potentially long lasting effects of writing manuscripts (Garrod et al., 2007; Levy, 2012).

Philosophical discussions about intersubjectivity routinely fail to mention the importance of objects and other features of the physical world. But as Tomasello noted above, a triadic interaction typically features an object as the vertex in a referential triangle involving infant and caregiver. It is the object or event which “joins” the individuals’ attention (Sinha and Rodríguez, 2008, 357) and it is “through participation in joint actions normatively structured around the use of artifactual objects…that the child finds an entry into the intersubjective realm of reasons for actions” (Sinha, 2009, 182). Peculiar objects, such as rolling balls or spinning tops, afford joint attention processes in non-trivial ways. But beyond such attention-grabbing toys, the material world is more than a canvas or blank slate for the play of human intersubjectivity. Enfield (2000, 42) observes that “representations are distributed across the ‘community of minds’ via coordinated focus on this mediating semiotic material—this may include gestures, proxemics, haircuts, people’s faces, melodies, cultural artifacts, odors, plants, animals, clothing, meteorological phenomena, among just about anything else that two people can coordinate attention on.” Intersubjectivity is thus dependent on “mediating structures” which include artifacts and the cultural practices that make sense of them (Hutchins, 1995). For instance, a person’s subjective perception of time is based on intersubjective notions of what time is and how it is measured, neither of which mean much without the calendars, clocks, and watches that people routinely put to use for purposes of interpersonal coordination (Williams, 2004).

The enaction of joint attention and shared intentionality, typically considered to be intersubjective phenomena, are also “interobjective.” The term interobjectivity came from Latour (1996, 240) who observed that “if you set yourself the task of following practices, objects and instruments, you never again cross that abrupt threshold that should appear, according to earlier theory, between the level of ‘face-to-face’ interaction and that of the social structure; between the ‘micro’ and the ‘macro’.” Latour suggests that a careful description of all the mediators involved—people, artifacts, and other structures—offers a window into processes operating across multiple time scales. Artifacts may serve as powerful repositories of symbolic meaning, but more than that, their built-in design and engineered affordances permit later generations and even historically discontinuous peoples to learn from and use these structures, often without explicit training (Malafouris and Renfrew, 2010; Hodder, 2012). These things are sometimes discussed as forms of “external memory” through which a society, wittingly or not, records its achievements for posterity (Donald, 1991; Meskell, 2005). And as important sources of information for social scientists, human artifacts are catalysts and precipitates of human interaction; artifacts are for the social sciences what fossils are for biology (Ehrlich and Ehrlich, 2008, 66). In artifacts we can trace the transformation over time of human interaction. Evolutionary theory has demonstrated the importance of studying fossils for working out the details of life’s history. Similarly, the study of artifacts may provide the sort of objective data necessary to unravel many of the key mysteries of cultural evolution (Basalla, 1988; Kirsh, 2010; Johannsen et al., 2014).

Our study differs from many earlier investigations of cooperative joint action by not only studying the intersubjective skills necessary for such interaction, but also “seeing through things,” in this case model cars, to derive conclusions about cultural processes as a whole. Too many cultural theorists forego the archeologists’ emphasis on material culture, but it is precisely in material culture that many of the conventions, representations, and “ideas” that others consider to be essentially private and abstract are to be found in concrete form (Sinha and Rodríguez, 2008, 364). By focusing on the dyad as our basic unit of analysis and by looking at LEGOs as mediators of cooperation, we have tried to overcome these limiting biases. In doing so, we foreground aspects of culture that have been previously understudied, namely its basis in skills for intersubjectivity *and* interobjectivity.

### GOALS OF THE STUDY

We consider our study and its results to be a “proof of concept.” This study presents methods for discerning, and quantifying, schema-like intersubjective understandings in material form. By designing experimental tasks that require pairs or groups to act together toward achieving—via LEGOs—a materialization of shared features of the environment (like CARS), concepts are transformed into percepts. This approach was inspired by recent work in cognitive science that looks to action and interaction for insights about human cognition (Rogoff, 1990; Varela et al., 1992; Hutchins, 1995; Goodwin, 2000; Noë, 2004; Stewart et al., 2010; Knoblich et al., 2011; Dale et al., 2014). In fact, the notion of the schema ought to be conceived as one feature in a much larger picture of human interaction. For schemas—just like words, phrases, and behaviors—only come about through the developmental processes that underlie human capacities in general, which derive from the fusion of our skills for intersubjectivity with our skills for interobjectivity.

## MATERIALS AND METHODS

The data below derived from a larger project investigating human interaction. Here, we present an analysis of the products of those interactions (i.e., the model cars built by the pairs of participants). The analysis of interaction measures is presented in additional publications based on the study (Mitkidis et al., in review; Wallot et al., in review).

### PARTICIPANTS

A total of 74 participants from Aarhus University participated in the experiment (average age: 23.5 years SD = 3.5 years) and were randomly assigned to pairs. Using standardized forms in the subjects’ native language, the pairs were instructed to cooperate in the construction of model cars using LEGO building bricks. The experiment lasted 75 min. At the end of the experiment participants were compensated with 350 DKK (≈47 EUR). The protocol was reviewed and approved by the Ethics Committee for Region Midtjylland, Denmark. All participants signed a written informed consent form.

### PROCEDURE

**FIGURE 2:**
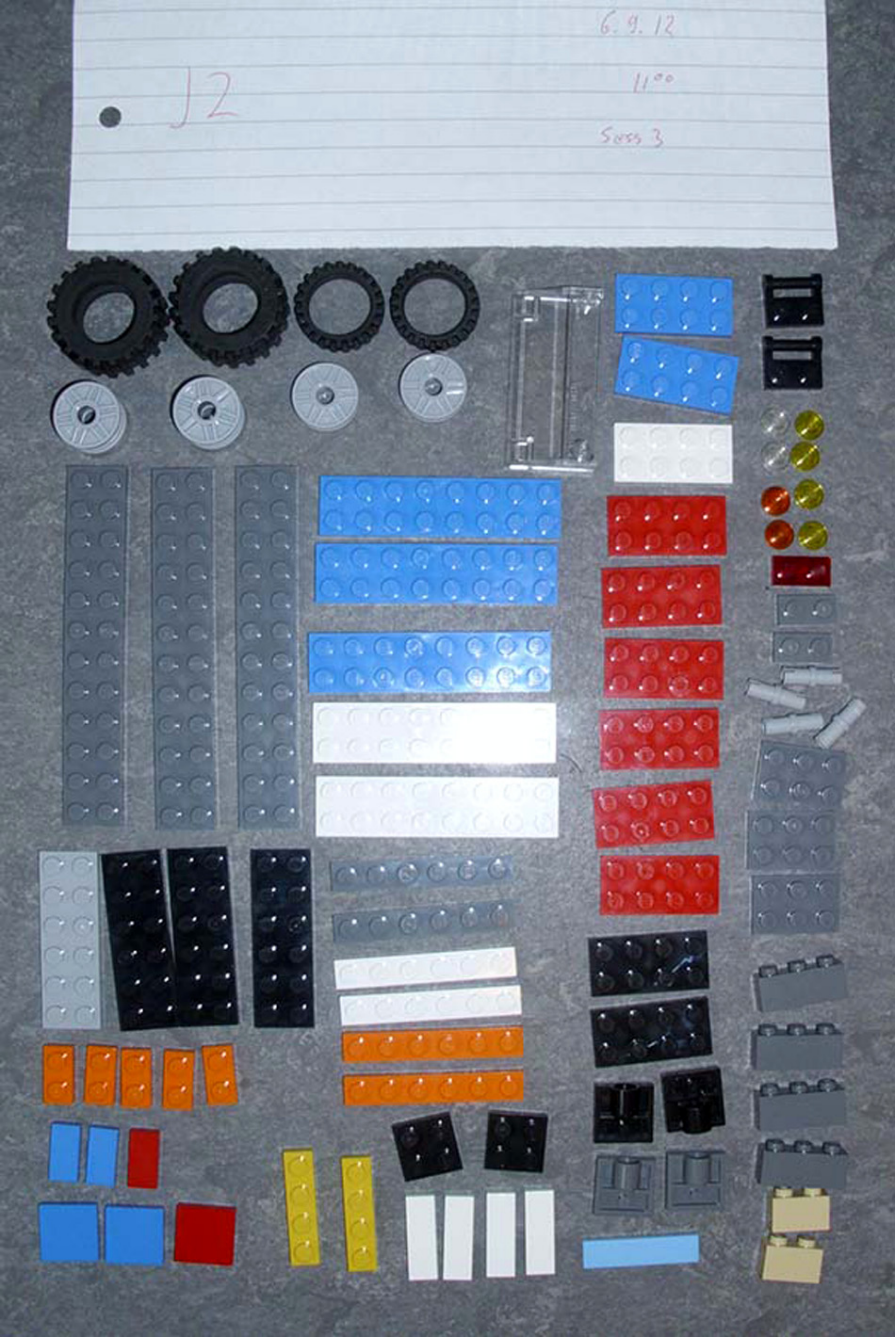
**A disassembled car model**.

The 37 pairs of participants used LEGO building bricks to construct model cars during four consecutive 10 min sessions. At the beginning of each building session, subjects were given a new box of LEGOs which contained the same building bricks present in every other session. Also, participants were given different instructions on how to go about building a car together during each session. The order in which the instructions were given was randomized for each pair of participants. Subsequent data analysis revealed that neither the modes of interaction (EC, TT, and HC) nor their order ended up being salient since the results described below demonstrate very strong carry-over effects from earlier to later sessions; if anything, the modes of interaction might have worked against this effect. Additional publications based on this study utilize results based on these modes of interaction and discuss their significance (Mitkidis et al., in review; Wallot et al., in review). At the end of each session, the model car and remaining LEGOs were removed from the room and the car was later photographed, both as an assembled model as well as a set of disassembled building bricks (see **Figure 2**). Each car’s pieces were counted and categorized using a unique identifier for the type and color of each LEGO brick.

## RESULTS

To evaluate similarity between any two cars, the number of different pieces they shared in common was calculated. Afterward, the number of common pieces was divided by the overall number of different pieces used in both cars to account for the fact that bigger cars will tentatively show greater overlap of component pieces by chance alone.

**FIGURE 3:**
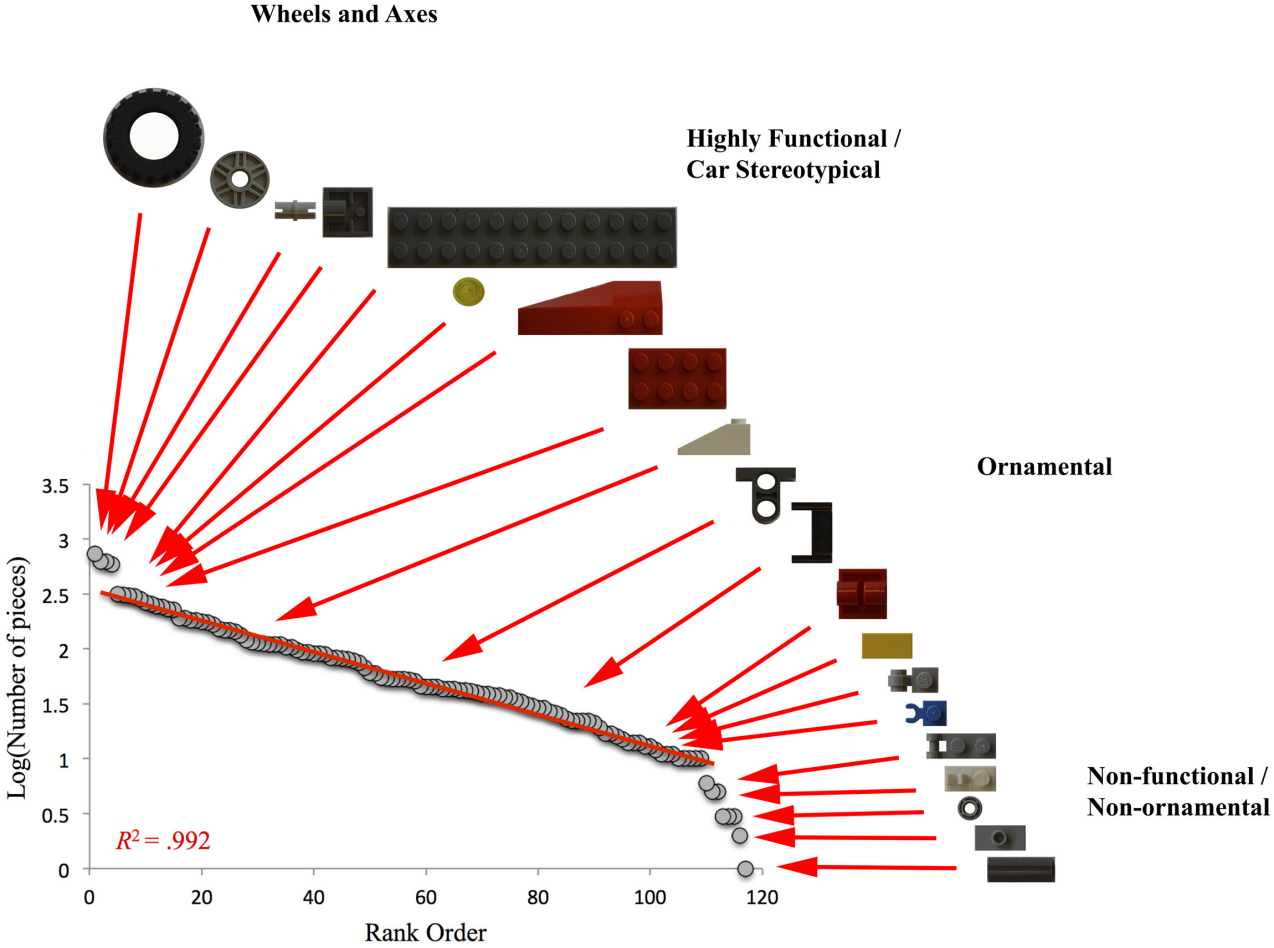
**Plot of the logarithm of number of pieces vs. the rank-order of pieces.** The middle part of the distribution is characterized by a strong relationship between functional/stereotypical vs. ornamental pieces. The front-end of the distribution marks fundamental, indispensable pieces (wheels and axes), while the back-end features increasingly non-functional, non-ornamental pieces.

To evaluate whether there was an overarching pattern across all pairs that reflected participants’ understanding of the concept of a car, rank-order distribution was constructed using all pieces from all cars. As can be seen in **Figure 3**, the number of pieces that were used to build cars scaled logarithmically to the rank order of pieces (*R*^2^ = 0.992) with exceptions at the front- and back-end of the distribution; very frequently and very infrequently occurring pieces deviated from this relationship. An inspection of these deviations revealed that the very frequently occurring pieces were wheels, hubs, and axes; arguably indispensable components of a car. The very infrequently used pieces seemed to be largely non-functional pieces that were neither necessary nor typical of cars and possessed little in the way of aesthetic or ornamental quality (see examples in **Figure 3**).

The broad, logarithmic distribution of pieces in between seem to fall on a continuum of highly functional (such as larger plates used to construct the chassis of a car) and highly stereotypical pieces (such as round, transparent pieces that typically served as car lights) on the high-frequency end, and increasingly non-functional pieces on the low-frequency end.

**FIGURE 4:**
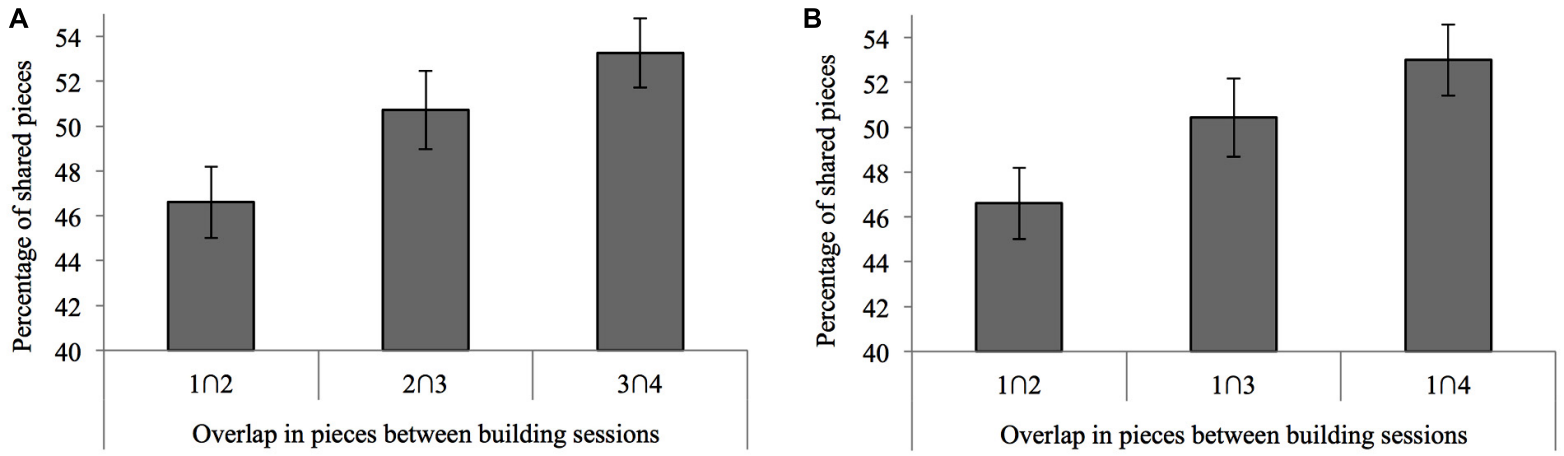
**Overlap of pieces between cars constructed in consecutive sessions (A) and overlap of pieces between the car built in the very first session compared to cars built in all subsequent sessions (B).** Cars constructed in later sessions showed an increasingly greater overlap with their predecessors, *and* with the very first models.

To investigate how cars developed across sessions, we investigated the average carry-over effect in pieces from one car to the next. The similarity between consecutively built cars increased from session to session (see **Figure 4A**); consecutive cars shared a greater and greater percentage of the same kinds of pieces [*F*(2,104) = 6.84, *p* = 0.002, η = 0.116]. Interestingly, there was also an increasing influence of the first model on consecutively built models, as models constructed in subsequent sessions shared an increasing amount of pieces with the *very first* model car built [*F*(2,104) = 6.31, *p* = 0.003, η = 0.108], as demonstrated in **Figure 4B**.

**FIGURE 5:**
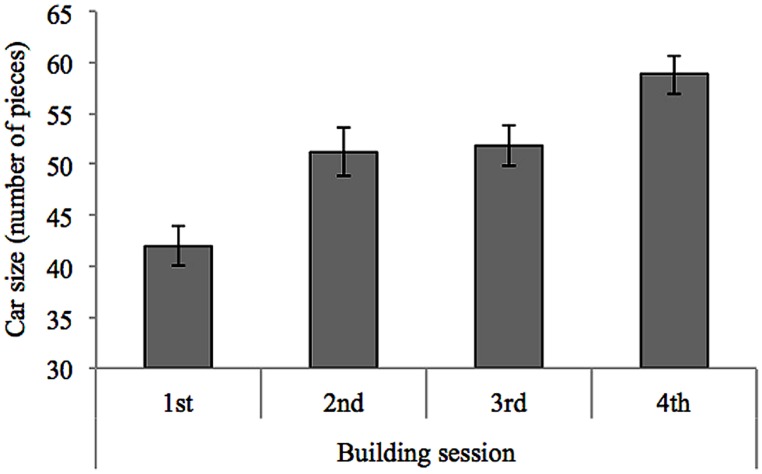
**Car size as a function of building session.** In successive building sessions, the car size, as measured by number of pieces, increases.

To investigate the rates of productivity across different building sessions, we calculated the size of each car (i.e., the number of its component pieces) and subjected the measure of car size to a repeated measures ANOVA with the factor session number (1, 2, 3, 4). As can be seen in **Figure 5**, cars grew bigger across the building sessions [*F*(3,156) = 18.80, *p* < 0.001, η = 0.266].

**FIGURE 6:**
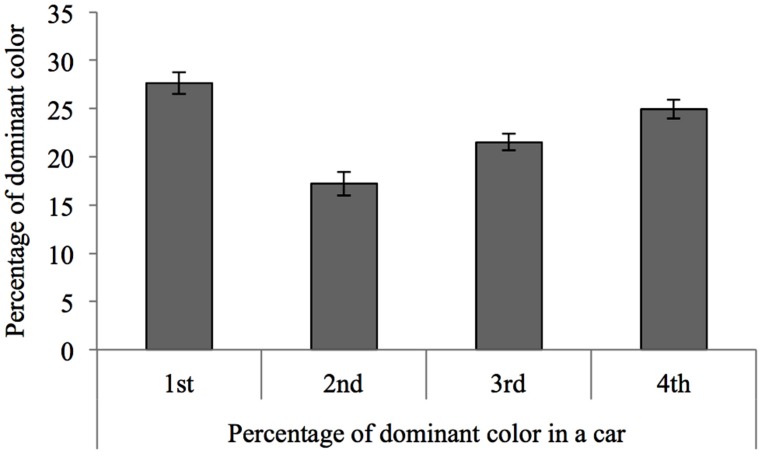
**Proportion of the dominant color in a car model as a function of building session.** The prominence of the dominant color in a car was strongest in the first building session and dropped from the first to the second session, only to steadily increase across the remaining three sessions.

To investigate how design features changed across building sessions, we calculated the dominant color used in the four building sessions by each pair of participants. This was done by calculating the percentage of LEGOs within each color category for each car, and then summing that percentage across all four cars built by each pair. We then investigated how the percentage of the dominant color changed across the four building sessions, subjecting the percentages to a repeated measure ANOVA with the factor session number (1, 2, 3, 4). As shown in **Figure 6**, the proportion of the dominant color was strongest in the first model and dropped off from session 1 to 2, but then increased steadily from sessions 2 to 4 [*F*(3,156) = 16.27, *p* < 0.001, η = 0.238].

**FIGURE 7:**
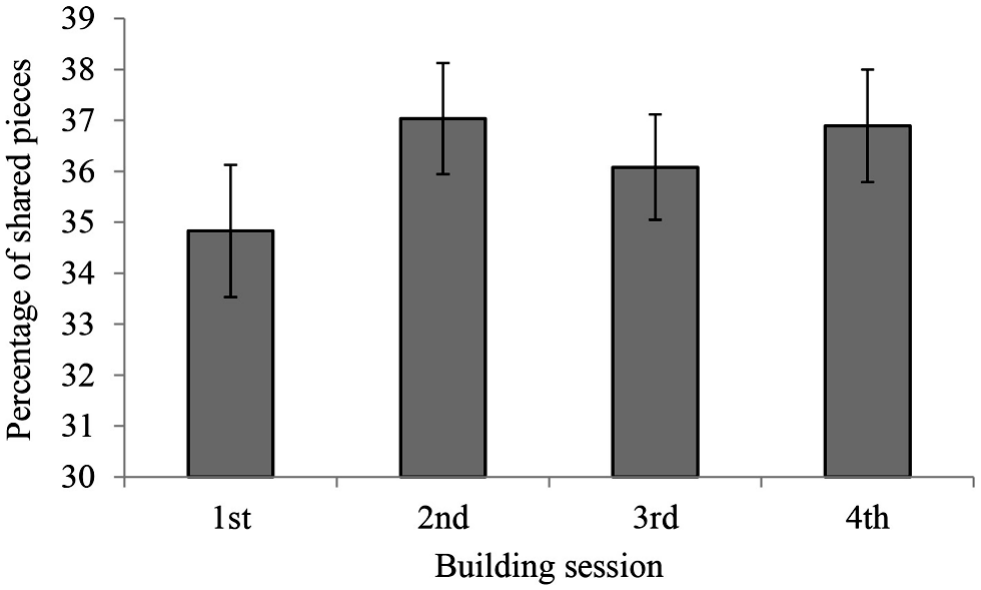
**Overlap of pieces between cars constructed by different pairs, but in the same session.** The similarity of cars within each session, quantified as the average number of pieces shared, did not differ as a function of building session.

While most of the aforementioned measures refer to patterns derived from within-pair comparisons across the four sessions, we also performed a between-pairs comparison of the overlap in LEGOs for sessions 1, 2, 3, and 4. As shown in **Figure 7**, the similarity of cars within each session, quantified as the average number of pieces shared, did not differ as a function of building session. To investigate the diversity of cars across sessions, we calculated the average overlap of pieces between all the cars constructed in each session [*F*(3,204) = 0.79, *p* = 0.502].

## DISCUSSION

As demonstrated in **Figure 3**, all car models featured many things in common. This almost certainly derives from the culturally mediated schemas participants share. Coming into the experimental setup with similar schemas exerts non-trivial influences on behavior since it greatly accelerates coordination among participants (e.g., they do not have to puzzle over what the word for car means or what a car should basically look like). Moreover, these shared schemas immediately reduce the possibilities given the large set of LEGOs; since individual building bricks are more or less important for constructing a model car, the actual set of LEGOs and their combinations far exceeds the usable set for accomplishing the task.

Translating the basics of car design into a LEGO model posed no real challenge for the pairs. They are heirs of a technological culture that worked out the basics of wheeled transport over many centuries. For example, cars cannot be built in such a way that two of their wheels roll in one direction while the other two roll perpendicular to that direction. Participants, because of the schemas they shared, did not need to engage in fruitless experiments regarding the alignment of wheels or hundreds of other possibilities that run counter to the basic template of a car; history had accomplished this work already. Perhaps they did not realize it, but all participants came into the experimental setting with all the know-how they required to build model cars from the very first building session. This is a significant point since people in other times and places would have no such knowledge, individually or collectively. It is because of this simple fact that an experiment like this can capture something meaningful about culture.

In constructing their first model, pairs negotiated significant coordination costs—they needed to learn how to successfully work with each other in achieving the task—that, once paid, could be reliably recaptured in each successive building session by working together in similar ways and producing a model that basically conformed to the prior models they had already produced. Successful coordination became increasingly predictable by adhering to designs that reified their prior coordination patterns. Car designs became more and more standardized across sessions, but they also *grew* from one session to the next. This increase in the number of pieces used for each car demonstrates something like a “ratchet effect” (Tomasello, 1999a, 37–41) in that the efficiencies of adopting conventions established in earlier sessions freed up resources (particularly time) for additional modifications in later sessions. Tomasello describes the ratchet effect as the ability, peculiar to humans alone, to faithfully learn and preserve innovations over time, and generations, which permits additional modifications to accumulate. This human capacity is ratchet-like not only because it slowly cranks things upward in complexity, but also because it prevents slippage that might cause innovations to be dropped (i.e., lost or forgotten; Tan and Fay, 2011). The kind of imitative learning that already begins to show up in triadic interactions leads to the “cumulative cultural complexity” that defines human culture; it is a form of inheritance that ties human bodies and minds to their artifacts, all of which have “cultural histories” (Tomasello, 2006, 206). As Tomasello (2006, 205) notes: “…none of the most complex human artifacts or social practices—including tool industries, symbolic artifacts, and social institutions—were invented once and for all at a single moment by any one individual or group of individuals. Rather, what happened was that some individual or group of individuals first invented a primitive version of the artifact or practice, and then some later user or users made a modification, an improvement, that others then adopted perhaps without change for many generations, at which point some other individual or group of individuals made another modification, which was then learned and used by others, and so on over historical time.” Tomasello concludes that just a few basic, though momentous, abilities which distinguish us from our nearest kin, the chimpanzees, were required for the development of human culture which he sees as our ability to create “history.” And history is not simply *having* a past, but the intentional *preservation* of the past—through memories, actions, and objects—so that it may have relevance for the present and future.

Given that participants began each session with the identical set of building bricks, it might be expected that they would produce four unique models. After all, the number of LEGO bricks used for an average car model produces immense combinatorial possibilities. However, as seen in the results, this was not the case. Others might expect that because participants’ schemas about cars are so similar, the pairs might find it most efficient to employ a “status quo” bias (Kahneman et al., 1991), essentially producing the same model again and again. As can be seen in the results, though, this is far from the stepwise progressions exhibited in the actual comparisons. The data revealed a surprising pattern in the selection of building bricks as well as features of car design across consecutive building sessions. The model in each later session demonstrated an increasing reliance on the model which immediately preceded it. Additionally, the very first model served an increasingly important role as a design template in each later session. As expressed by the cars themselves, each pair of participants seems to have consolidated their schematic representations of LEGO model cars, so that they became increasingly convinced what a LEGO car “ought” look like as they proceeded from one session to the next.

### THE PERSISTENCE OF MEMORY

When looking over the results, a set of stepwise progressions shows up across numerous measures. We identify these patterns as “path dependence” (David, 1985; Liebowitz and Margolis, 1995; Garud and Karnøe, 2001b) demonstrative of rapid conventionalization. Path dependence refers to the ability of influences from the past, usually near the beginning of a phenomenon, to strongly constrain aspects of its future. This often occurs even when the early conditions have little functional relevance for later conditions. Garud and Karnøe (2001a, 4) describe how “phenomena are sensitive to small differences in the underlying sequence of events” such that “a steady accumulation of small differences can result in the technological field locking onto a trajectory.” In broad terms, path dependence exhibits the persistence of past states in future states and has often been discussed using the truism “history matters” (North, 1990, 100; David, 2001).

The notion of path dependence has been influential in economic theory, where scholars have often invoked it to explain inefficiencies that endure in spite of seemingly superior alternatives (Liebowitz and Margolis, 1995). Examples from technological history have played an important role in demonstrating the power of path dependence. David (1985) described how early models of typewriters required organizing the keyboard using the QWERTY layout that has dominated ever since. However, the mechanical reasons for implementing this format ceased to be relevant a short time later, as new mechanisms were introduced. And, of course, these mechanical constraints have no relevance for computer keyboards which use an entirely different implementation to link keystroke inputs to graphic outputs. The QWERTY layout has endured in spite of reasoned alternatives at the time and greatly superior alternatives at later times. In the 1930s, a pair of education professors by the name of Dvorak and Dealey developed a keyboard configuration that permits users to type much faster while also reducing errors and strain (see Noyes, 1983). Nevertheless, the ready availability of QWERTY typewriters ensured that the majority of typists would learn using this layout and the fact that the majority of typists continued to learn the QWERTY format ensured that manufacturers would continue producing such machines in greater and greater quantities over time.

**FIGURE 8:**
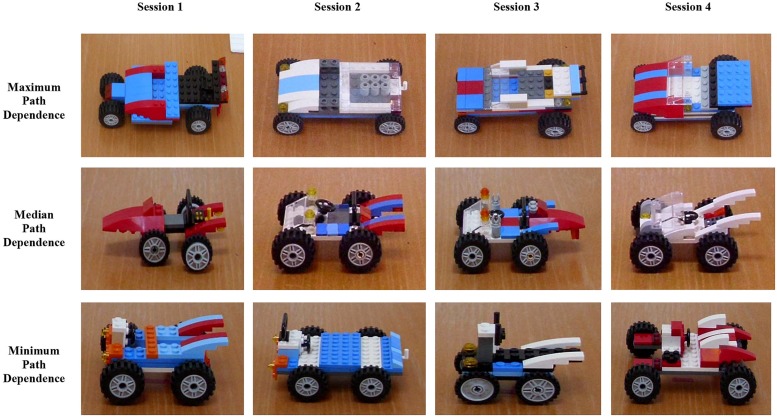
**Sets of cars demonstrating varying degrees of path dependence**.

This example highlights how path dependence relies on “positive feedback,” the amplification of an effect by its influence on the processes which give rise to it. The fact that there is a superior alternative to the QWERTY layout and that rational consumers ought to select the superior format over the inferior one—as many economic models would predict—is not, in fact, what occurred. Mechanical constraints at an early stage of development necessitated a particular layout which has dominated ever since, in spite of better alternatives. According to adherents of the path dependence model, this suggests that history can trump powerful competing principles: “History then is the tool to understand what rationality and efficiency do not explain, that is, the random sequence of insignificant events that are not addressable by economic theory” (Liebowitz and Margolis, 1995, 17–18). As is evident in this case, as well as in many other instances of history—from the demise of the dinosaurs due to a stray meteor to the discovery of the American continents by sailors searching for a quicker route to Asia—*contingent* events, that is, events which might have transpired in some other way, often change things in ways that cannot be foreseen, even using the best scientific models at our disposal. In similar fashion, participants had tremendous freedom in developing their first car models but the relatively arbitrary forms they settled upon exerted downstream influences on all their later models, an effect very much like path dependence (see **Figure 8**).

Relating these findings to evolutionary theory, Stephen Jay Gould’s book, *Wonderful Life: The Burgess Shale and the Nature of History*, offers a provocative interpretive framework. In the book, Gould investigates the significance of the “Cambrian explosion,” a geological period that began around 540 million years ago, for the theory of evolution. In just 60 million years, life went from a small variety of relatively simple organisms to a huge diversity of complex organisms; almost all animal phyla (“the fundamental ground plans of anatomy”) developed in this period and very few new ones have come into being in the 500 million years afterward. The most remarkable finding, according to Gould, is not that this proliferation occurred, but that animals on Earth today evolved from only a fraction of those which existed during this prolific era. Instead of a continued diversification of organisms, as exhibited during the Cambrian explosion, a small sample from that time served as ancestors for all later life. Gould (1989, 47) notes that “the later history of life proceeded by elimination, not expansion. The current earth may hold more species than ever before, but most are iterations upon a few basic anatomical designs.” Much impressed with the odd, often fantastic, anatomical varieties present in the Cambrian period (and preserved in the Burgess Shale), Gould (1989, 47) observes that “later history is a tale of restriction, as most of these early experiments succumb and life settles down to generating endless variants upon a few surviving models.” Gould argues that once a basic form proves to be successful it begins to reproduce rapidly and its variations become increasingly subtle over time. There are fewer and fewer grand design changes of the sort that would revoke the “ground plans of its anatomy” and potentially lead to a new phylum. What is common to both the path dependence literature, particularly in relation to technology, and to evolutionary theory is that big innovations early on establish a path which all later members of the type follow. Whether it be the dominance of the QWERTY layout over and against novel keyboard layouts a short time later or the hegemony of a subset of phyla for more than 500 million years, a principle of evolution seems to be that basic forms established early on consolidate their hold and prevent interloper designs from entering their niche. This process reduces diversity of form but accelerates increasingly specific processes of optimization.

Just as Gould might have predicted, our results demonstrate the inordinate importance of the first car model for shaping later models. While the nature of the experimental setup provides participants with a set of conditions to produce four novel designs, the opposite, in fact, occurs. Reflecting on the evolutionary process, Gould (1989, 321) notes how “little quirks at the outset, occurring for no particular reason, unleash cascades of consequences that make a particular future seem inevitable in retrospect. But the slightest early nudge contacts a different groove, and history veers into another plausible channel, diverging continually from its original pathway. The end results are so different, the initial perturbation so apparently trivial.” Instead of evolutionary processes completely determining the nature and scope of life, he asserts “history as the chief determinant of life’s directions” (1989, 288). Similarly, each pair’s first car model, that first concatenation of arbitrary design decisions and brick selections, served as a design template for all later building sessions, which ended up as variations upon a theme. And just as with the distinctive phyla established during the Cambrian, car designs made by *different* pairs showed no convergence (see **Figure 7**). This seems to indicate that there were no constraints or attractors based on function or optimality that would cause all pairs to converge toward an “ideal” design. Instead, it is as if those arbitrary first designs established distinctive channels which, while running concurrently and in parallel, did not have any particular aim toward which they might evolve.

Stuart Kauffman (1995, 195), a theoretical biologist and complexity theorist, and Gould are in agreement regarding the general pattern of life since the Cambrian explosion, namely that once “species with a number of major body plans sprang into existence, this radical creativity slowed and then dwindled to slight tinkering. Evolution concentrated its sights closer to home, tinkering and adding filigree to its inventions.” This reduction in basic diversity relates to the amplification of “conflicting constraints” as organisms become increasingly “locked in” to their fundamental anatomy (1995, 199–201) and as all evolving life becomes more and more competent for its niche so that interlopers face greater competition.

Kauffman (1995, 202) takes this “Cambrian pattern of diversification” even further, believing it to be exhibited in a wide range of complex phenomena, including technological evolution: “...given a fundamental innovation—gun, bicycle, car, airplane—it appears to be common to find a wide range of dramatic early experimentation with radically different forms, which branch further and then settle down to a few dominant lineages.” To be clear, neither Gould nor Kauffman argue against the increase of *overall* diversity through evolutionary processes, but posit a reduction in the diversity of *basic* forms, what corresponds to the level of phyla in biological taxonomy. Subsequently, increased diversification happens at lower taxonomic ranks, particularly through speciation. Reviewing his juxtaposition of the Cambrian explosion with technological evolution, Kauffman (1995, 205) concludes: “the parallels are striking, and it seems worthwhile to consider seriously the possibility that the patterns of branching radiation in biological and technological evolution are governed by similar general laws…tissues and terra-cotta may indeed evolve in similar ways. General laws may govern the evolution of complex entities, whether they are works of nature or works of man.” Kauffman’s assertion that a Cambrian pattern of diversification may be applicable to technological evolution would seem to be exhibited in the results of this joint action study. The first car established something like a “phylum” which consolidated in each successive session. This pattern seemed to apply both to the LEGO bricks selected as well as the dominant color participants settled upon. The results seen in this study may exhibit larger dynamics of cultural evolution, a set of dynamics that fall in line with the phenomenon called path dependence. And while the warrant is tentative, similar dynamics may also shape complex phenomena as diverse as anatomical structures and the evolution of technology.

## CONCLUSION

Few would argue against Tomasello’s description of the ratchet effect leading to “cumulative cultural complexity,” but most would assume this to mean increasing diversification as time goes forward. The argument here is that the cumulative complexity of culture occurs in a subtle fashion: for any cultural innovation, experiments in basic form lead soon thereafter to processes of reduction and elimination as a dominant path is established. From that moment onward, increasingly small, and gradual, modifications reiterate the basics of the original form.

Given the results of this “proof of concept” study, it would seem that applying evolutionary theory to the study of culture is a generative exercise. And this would seem to be true in spite of the fact that the phenomena in question, biological transformation over time and cultural transformation over time, operate on qualitatively different “kinds.” Biology and culture are continuous, but they are clearly not the same thing; transformation over time, however, refers to a set of processes that may well apply to a wide range of phenomena. In pursuit of this, we have utilized ideas about path dependence in our analysis of the products of joint action. A prominent pattern across many phenomena is a reduction in the diversity of basic forms over time. Based on these findings, it is reasonable to conclude that solutions to invariant tasks and challenges need not be endlessly novel, thus draining energy and resources from other tasks and challenges; an earlier solution that has already proven to be satisfactory is the foundation upon which subtler optimizing processes can set to work. An additional reduction in variability derives from shared schemas that facilitate intersubjective as well as interobjective coordination. The possession and use of schemas means that we approach a task with many ideas about the world shared in common. Even though these ideas greatly constrain potential variability, their usefulness in promoting coordination enhances overall efficiency. As present and subsequent experience can be made to “more or less” accommodate prior expectations, and update those expectations, the adjustments necessary to succeed in the present are greatly reduced in both time and complexity.

## Conflict of Interest Statement

The authors declare that the research was conducted in the absence of any commercial or financial relationships that could be construed as a potential conflict of interest.

## Footnotes

1 Scaffolding, in this case, refers to the alteration of the environment, and of the instruction itself, to meet the learner’s needs. Like the use of scaffolding in construction, the notion is that a set of temporary supportive practices and artifacts are put into place to facilitate the construction process, until the project is self-supporting or completed. Wood et al. (1976) were among the first to use the term in relation to teaching and learning.
